# Optimized decellularization protocol including α-Gal epitope reduction for fabrication of an acellular porcine annulus fibrosus scaffold

**DOI:** 10.1007/s10561-017-9619-4

**Published:** 2017-03-24

**Authors:** Lien-Chen Wu, Yi-Jie Kuo, Fu-Wen Sun, Chia-Hsien Chen, Chang-Jung Chiang, Pei-Wei Weng, Yang-Hwei Tsuang, Yi-You Huang

**Affiliations:** 1 Institute of Biomedical Engineering, College of Engineering, College of Medicine, National Taiwan University, No. 1, Sec. 1, Jen-Ai Road, Taipei, Taiwan; 2 Department of Orthopedics, Shuang Ho Hospital, Taipei Medical University, Taipei, 23561 Taiwan; 3 Department of Orthopedics, Taipei Medical University Hospital, Taipei, 110 Taiwan; 4 Department of Orthopaedics, School of Medicine, College of Medicine, Taipei Medical University, Taipei, 11031 Taiwan

**Keywords:** α-Gal epitope, Annulus fibrosus, Intervertebral disc, Decellularization, Tissue engineering

## Abstract

Recent advances in tissue engineering have led to potential new strategies, especially decellularization protocols from natural tissues, for the repair, replacement, and regeneration of intervertebral discs. This study aimed to validate our previously reported method for the decellularization of annulus fibrosus (AF) tissue and to quantify potentially antigenic α-Gal epitopes in the decellularized tissue. Porcine AF tissue was decellularized using different freeze–thaw temperatures, chemical detergents, and incubation times in order to determine the optimal method for cell removal. The integrity of the decellularized material was determined using biochemical and mechanical tests. The α-Gal epitope was quantified before and after decellularization. Decellularization with freeze–thaw in liquid nitrogen, an ionic detergent (0.1% SDS), and a 24 h incubation period yielded the greatest retention of GAG and collagen relative to DNA reduction when tested as single variables. Combined, these optimal decellularization conditions preserved more GAG while removing the same amount of DNA as the conditions used in our previous study. Components and biomechanical properties of the AF matrix were retained. The decellularized AF scaffold exhibited suitable immune-compatibility, as evidenced by successful in vivo remodeling and a decrease in the α-Gal epitope. Our study defined the optimal conditions for decellularization of porcine AF tissues while preserving the biological composition and mechanical properties of the scaffold. Under these conditions, immunocompatibility was evidenced by successful in vivo remodeling and reduction of the α-Gal epitope in the decellularized material. Decellularized AF scaffolds are potential candidates for clinical applications in spinal surgery.

## Introduction

Intervertebral disc herniation is a common cause of severe lower back pain and disability. The available surgical treatments, including discectomy and spinal fusion, are primarily palliative and are meant to eliminate pain in the short term. They neither repair the disc nor restore the normal biological and mechanical properties of the spine. Such procedures can limit mobility and may even further alter the biomechanics of the spine, leading to further degeneration of adjacent segments. Thus, new strategies for treating damaged intervertebral discs are needed. Recent advances in tissue engineering present potential new strategies to repair, replace, or regenerate degenerative discs (Mercuri et al. 2011; Schek et al. 2011; Yang et al. 2009).

The intervertebral disc (IVD) provides a cushion between the vertebrae, protecting them from compression and other stress. Each disc comprises a soft center (the nucleus pulposus) surrounded by a tough outer wall of collagen and proteoglycans (the annulus fibrosus). Tears and fissures of the annulus fibrosis (AF) due to decreased integrity of the extracellular matrix or traumatic injury result in protrusion of the nucleus pulposus (NP). The low vascularity and low cellularity of the AF limit its intrinsic healing capacity, as with articular cartilage (Bron et al. 2009; Mwale et al. 2011). Treatments to repair such herniation without discectomy require AF repair that is suitable to withstand the intradiscal pressure (Guterl et al. 2013). Direct suture of the AF is technically very demanding due to limited space and potential injury to the proximal neurological structures (Guterl et al. 2013). Implants for closing the damaged AF are commercially available, but these do not prevent AF degeneration or maintain the biological AF structure in ensuing years (Bron et al. 2009; Chan and Gantenbein-Ritter 2012). Materials that approximate the extracellular matrix (ECM) of the AF, a key contributor to its high strength, are thus highly attractive for such repair.

One tissue engineering strategy for developing biomaterials is to decellularize xenogeneic tissues by removing immunogenic cells. This method has been successfully used to develop biomaterials for use in cartilage, the meniscus, ligaments, and tendons, with impressive results (Gilbert et al. 2006, 2009; Stone et al. 2007; Wu et al. 2014; Xu et al. 2014). Xenogeneic and allogeneic cellular antigens are recognized as foreign by the host, thereby inducing an inflammatory response or an immune-mediated rejection of the tissue. However, ECM components such as collagens, laminins, and polysaccharides, are highly conserved among species and are tolerated well even by xenogeneic recipients (Gilbert et al. 2006), (Gilbert 2012). The goal of a decellularization protocol is to remove all cellular and nuclear materials to prevent immune and inflammatory reactions while minimizing any adverse effect on the composition, biological activity, and mechanical integrity of the remaining ECM. When properly processed to remove cellular antigens that can induce immune rejection without damaging the ECM, ECM scaffolds can serve as potent sources of cues that promote constructive remodeling of tissue after injury or degeneration.

We previously reported a preliminary method for decellularizing porcine AF to yield a biological scaffold for use in human AF defect substitution (Wu et al. 2014). Our method produced a scaffold with low DNA content that retained most of the original biological composition and properties, with some loss of glycosaminoglycan (GAG). The present study aimed to manipulate several protocol variables in this method to determine conditions that would maximize retention of the scaffold components collagen and GAG while minimizing the DNA content. A secondary objective was to determine the biochemical and biomechanical properties of the acellular AF scaffold and to preliminarily evaluate its biocompatibility using α-Gal epitope quantification, in vitro cytotoxicity assays, and an in vivo immuno-compatibility study.

## Materials and methods

### Tissue harvest

This study used xenogeneic tissue to facilitate tissue sourcing. Fresh porcine lumbar spines were obtained *en bloc* from a local abattoir (Taoyuan County, Taiwan) within 2 h post-mortem. The AF tissue was harvested from the IVD by gently excising and washing in phosphate-buffered saline (PBS) to remove excess blood. To preclude contamination by the surrounding tissue and NP, the outermost and innermost layers of the AF were carefully removed. Samples were then placed in neutral buffered formalin for histologic analysis or frozen and stored in PBS-moistened filter paper at −20 °C.

### Comparison of decellularization methods by biochemical assays

#### Protocol 1: Freeze–thaw in −80 °C versus liquid nitrogen (−196 °C)

This decellularization method was a modification of the method of Booth et al. (2002), Stapleton et al. (2008). Briefly, the AF samples were randomly divided into two groups (n = 6) and decellularized by exposing the tissue to freeze–thaw. Porcine AF was either frozen at −80 °C for 22 h (Group A), or in liquid nitrogen (−196 °C) for 22 h (Group B), and then thawed in an incubator at 37 °C for 2 h. Samples were then incubated with agitation in hypotonic buffer (10 mM tris–HCl, pH 8.0) with 0.1% EDTA and protease inhibitors (aprotinin 10 KIU/mL; Sigma, USA) at 37 °C for 24 h, then in 0.1% sodium dodecyl sulfate (SDS, Wako, Japan) at 45 °C for 24.

After washing, the samples were incubated in deoxyribonclease (DNase 50U/mL; Sigma, USA) and ribonuclease (RNase 1U/mL; Sigma, USA) in Tris buffer (50 mM tris–HCl, 10 mM magnesium chloride, and 50 mg/mL bovine serum albumin at pH 7.5) for 3 h at 37 °C with gentle agitation. The tissues were then washed three times in PBS at 37 °C for 8 h. Fresh porcine AF samples (controls) were stored at −20 °C. All AF samples were then evaluated by biochemical assay (sulfated glycosaminoglycan assay, hydroxyproline assay, and DNA assay, detailed methods are described later) to compare AF properties between tissues treated with and without decellularization.

#### Protocol 2: 0.1% SDS versus 1% Triton x-100

Freeze–thaw in liquid nitrogen was used, as it resulted in more effective decellularization than did freeze–thaw at −80 °C. The AF samples were randomly divided into C and D groups (n = 6), and all samples were freeze–thawed in liquid nitrogen and then incubated in a hypotonic buffer at 37 °C for 24 h. The group C samples were then decellularized in tris–HCl buffer containing 0.1% SDS, 0.1% EDTA, and 10 KIU/mL aprotinin at 4 °C for 24 h. The group D samples were decellularized in tris–HCl buffer with 1% Triton X-100 (Sigma, USA), 0.1% EDTA and 10 KIU/ml aprotinin at 4 °C for 24 h. The samples were washed and incubated with DNase and RNase in Tris buffer, washed for 8 h in PBS, and evaluated by biochemical assay as described in Protocol 1.

#### Protocol 3: Comparison between decellularization times of 24 versus 48 vs. 72 h using 0.1% SDS

Freeze–thaw in liquid nitrogen followed by treatment with 0.1% SDS showed better decellularization results compared to 1% Triton X-100, and was therefore selected as the optimal method. AF samples were randomly divided into 3 groups (n = 6/group), freeze–thawed in liquid nitrogen and then incubated in a hypotonic buffer at 37 °C for 24 h. The samples were then decellularized in tris–HCl buffer containing 0.1% SDS, 0.1% EDTA and 10 KIU/mL aprotinin at 4 °C for 24 h (group E), 48 h (group F), or 72 h (group G). The samples were washed, incubated with DNase and RNase in Tris buffer, washed for 8 h in PBS and evaluated by biochemical assay as described in Protocol 1.

### Optimal decellularization method

Based on our optimization data, we treated AF samples (n = 10) by freeze–thaw in liquid nitrogen, incubation in a hypotonic buffer at 37 °C for 24 h, and decellularization in 0.1% SDS, 0.1% EDTA and 10 KIU/mL aprotinin at 4 °C for 24 h. After washing, samples treated with DNase and RNase, stored, and assayed as described in the previous section.

### Biochemical assays

#### Sulfated glycosaminoglycan assay

The proteoglycan content of the tissue was determined by measuring the amount of sulfated glycosaminoglycans in the papain-digested tissue using the 1,9-dimethylmethylene blue (DMMB) dye binding assay and spectrophotometry (Farndale et al. 1986). The AF tissue specimens (n = 6) were first lyophilized (dry weight, 50 mg) and then digested in papain buffer (250 µL papain in PBS at pH 6.0 with 150 mM sodium chloride, 55 nM sodium citrate, 5 mM cysteine-HCl, and 5 mM Na_2_EDTA) at 60 °C for 12 h. The supernatant fluid was measured at 530 nm using chondroitin sulfate as a standard.

#### Hydroxyproline assay

The collagen content was quantified using a commercially available assay kit (hydroxyproline assay kit, BioVision, Milpitas, CA, USA) (Zhang et al. 2012). The AF specimens (n = 6) were first lyophilized and then hydrolyzed in 6 M HCl at 120 °C for 3 h and neutralized using NaOH. The hydroxyproline content was determined using the chloramine-T/Ehrlich’s reagent assay and spectrophotometry at 550 nm. The concentration of hydroxyproline was then calculated by interpolation from a hydroxyproline standard curve.

#### DNA assay

The residual cells were determined by DNA assay using the ReliaPrep gDNA Tissue Miniprep System (Promega, Fitchburg, WI, USA) (Rabelo-Goncalves et al. 2014). The DNA was extracted from both native and decellularized AF. Six AF specimens (wet weight, 25 mg) were used. The DNA was extracted using a DNA isolation kit and then quantified according to standard protocols by measuring the absorbance at 260/280 nm using a spectrophotometer (NanoDrop ND 1000; Thermo Scientific, Waltham, MA, USA).

### Histology

Tissue specimens (n = 5) were fixed in neutral buffered formalin for 48 h, then embedded in paraffin wax and sectioned at 6 µm thickness using a microtome. Samples were characterized histologically by hematoxylin and eosin staining (H&E, Bios Europe, Skelmersdale, UK) to evaluate cellular content and the AF structure and by Masson’s Trichrome Staining (MT staining) to visualize collagen distribution and orientation. Glycosaminoglycans (GAGs) were visualized by periodic acid-schiff (PAS) staining.

### Scanning electron microscopy (SEM)

Fresh-frozen AF and the decellularized AF scaffolds prepared by optimal decellularization method (OC) were harvested and fixed in 4% paraformaldehyde (wt/vol) for 2 days. The samples (n = 5) underwent sequential dehydration and were sputter-coated with gold (5 nm thick) to impart electrical conductivity. The specimens were then evaluated and imaged using SEM (JSM 5600, JEOL, Tokyo, Japan) to evaluate the ultra-structure of the AF surfaces.

### Compression (indentation) test

Compression tests were conducted using a Universal Testing Machine. Uni-axial compression tests were performed at room temperature (26 °C) and 60% relative humidity. The constant deformation rate was set at 0.1 mm/sec for all materials examined (n = 6). Force and deformation (changes in length) were recorded electronically, and the resulting stress–strain compression curves were constructed.

### α-Gal ELISA test sample processing

Native AF and decellularized AF scaffolds (OC) were thoroughly rinsed in ice-cold PBS (0.02 M, pH 7.0–7.2) for a brief washing and weighing before homogenization. The tissues were minced into small pieces and homogenized in a PBS using a glass homogenizer on ice. The resulting suspension was subjected to ultrasonication to further break down cell membranes and then centrifuged for 15 min at 1500×*g*. The supernatant was then removed and assayed as follows.

### Quantification of α-Gal content

The ELISA test for α-Gal was carried out as previously reported (Naso et al. 2011, 2013) using a commercially available assay kit (Alpha-Galactosyl ELISA Kit, BlueGene Biotech, Shanghai, China). Microtitre-plate wells pre-coated with antibody were loaded with 100 μL of supernatant derived from native AF tissue (n = 8) and decellularized AF tissue (n = 8). Conjugate (50 μL) was then added to each well, and the mixture was incubated for 1 h at 37 °C in darkness. The micro-titer plate was washed 5 times; after the final wash, the plate was inverted and blotted dry.

After drying, 100 μL of horseradish peroxidase substrate buffer was added to each well and incubated for 15 min at room temperature in darkness. Stop solution (50 μL) was then added to each well, and the Optical Density (OD) was immediately read at 450 nm using a micro-titer plate reader.

### Cytotoxicity of decellularized AF in a trans-well insert model

Samples of decellularized AF scaffolds (OC) were prepared and placed at the bottom of trans-well insert wells before cytotoxicity testing. The trans-wells used in this study were 24 mm in diameter, with a membrane pore size of 0.4 µm (Costar, Transwell, Corning, NY, USA). The trans-wells were placed onto 24-well culture plates, seeded with NIH3T3 fibroblasts (3 × 10^3^ cells/well), and incubated for 1 or 2 days. For each sample, 5 wells were prepared.

For controls, the cells were cultured in 24-well plates with trans-well inserts but without AF tissue. After co-culture, the insert wells were removed. Viability of the target NIH3T3 fibroblasts was measured using 3-(4,5-dimethylthiazol-2-yl)-5-(3-carboxymethoxyphenyl)-2-(4-sulfophenyl)-2H-tetrazolium (MTS, Promega) according to the manufacturer’s instructions.

### In vivo immuno-compatibility study

The host response to decellularized AF was assessed in 6 male Wistar rats (300–350 g), and all experiments were conducted according to the principles and procedures described by the Institutional Animal Care and Use Committee of National Taiwan University. Briefly, two non-contiguous coccygeal intervertebral discs were selected for the study. After anesthesia, a longitudinal incision was created over the tail region, and the posterior surface of the coccygeal intervertebral discs was exposed by anatomic dissection along the skin incision. Care was taken not to interrupt the outer annular fibers of the disc. A 1 × 1 mm rectangular excision was made through the annulus fibrosus and into the nucleus pulposus.

The two non-contiguous discs were delegated to the control group (box incision only) and the repair group, in which the box defect was implanted with decellularized AF scaffold (sterilized) and sutured using 6-0 non-absorbable polypropylene. In each group, rats were further divided into 2 subgroups, with 2 healing times, 7 and 14 days. The intervertebral disc levels of the annular incisions were randomized from animal to animal to diminish any level-specific differences. Upon euthanization, the discs were harvested and examined histologically for signs of inflammatory response (H&E staining) and ECM production (MT and PAS staining).

### Statistical analysis

All data are presented as the mean ± standard deviation (SD). Analysis of variance (ANOVA) was implemented to test differences between 3 or 4 groups, and independent *t*-tests were carried out to compare 2 groups. All statistics were two-sided, and *p* < 0.05 was considered statistically significant. Statistical analyses were performed using SPSS statistical software for Windows (version 22.0, IMB Corp., Armonk, NY).

## Results

### Comparison of decellularization methods by biochemical assays

The quantities of decellularized AF components under various freeze–thaw temperatures, detergents, and times are summarized in Table 1. The levels of ECM components were lower after decellularization in liquid nitrogen (−196 °C) than in control AF samples. However, samples decellularized with liquid nitrogen had a higher content of GAG, collagen and DNA compared to those decellularized at −80 °C.

**Table 1:** Results of biochemical assays of AF in decellularization

	GAG content (µg/mg)	Collagen content (µg/mg)	DNA content (ng/mg)
*Freeze*–*thaw temperatures*			
Fresh AF (n = 6)	107.07 ± 7.24	102.15 ± 9.93	46.31 ± 5.92
Freeze–thaw in -80 °C (n = 6)	94.03 ± 7.18^a^	93.62 ± 5.26^a^	10.38 ± 1.19^a^
Freeze–thaw in -196 °C liquid nitrogen refrigeration (n = 6)	99.57 ± 7.70^a,b^	101.75 ± 6.97^a,b^	12.03 ± 2.52^a,b^
*Detergents*			
Fresh AF (n = 6)	150.97 ± 4.60	117.68 ± 7.40	73.22 ± 5.43
Decellularized in SDS (n = 6)	142.85 ± 4.9^a^	108.58 ± 11.58^a^	12.34 ± 3.14^a^
Decellularized in Triton X-100 (n = 6)	127.08 ± 8.45^a,c^	100.79 ± 10.86^a,c^	25.59 ± 7.04^a,c^
*Decellularization time*			
Fresh AF (n = 6)	96.09 ± 2.88	120.94 ± 4.33	46.31 ± 5.92
Decellularization for 24 h (n = 6)	82.77 ± 6.17^a^	109.72 ± 2.96^a^	11.07 ± 2.17^a^
Decellularization for 48 h (n = 6)	47.49 ± 6.25^a,d^	94.18 ± 3.28^a,d^	9.16 ± 2.11^a^
Decellularization for 72 h (n = 6)	14.44 ± 2.90^a,d,e^	89.80 ± 5.85^a,d,e^	4.23 ± 1.34^a^
*Optimal decellularization methods (ODM)*			
Fresh AF (n = 10)	121.63 ± 5.66	153.63 ± 17.14	63.55 ± 3.71
Optimal decellularization methods (n = 10)	114.77 ± 10.61	151.73 ± 12.62	9.10 ± 2.20^a^

Data are presented as mean ± standard deviation, and tested by analysis of variance for comparisons among 3 or 4 groups, or by independent *t* test for comparison between 2 groups

*AF* annulus fibrosus, *GAG* glycosaminoglycans, *SDS* sodium dodecyl sulfate

^a^Significantly different from Fresh AF group, *p* < 0.05

^b^Significantly different from −80 °C group, *p* < 0.05

^c^Significantly different from SDS group, *p* < 0.05

^d^Significantly different from 24 h group, *p* < 0.05

^e^Significantly different from 48 h group, *p* < 0.05

More GAG and collagen content was preserved by decellularization in 0.1% SDS than in Triton X-100. However, the DNA content in the 0.1% SDS group was lower than in the Triton X-100 group and was therefore superior to Triton X-100 for AF decellularization.

The DNA content was significantly lower after 24 h of decellularization and effectively removed after 72 h. However, extension of the decellularization time resulted in greater losses of GAG and collagen. Thus, the ideal decellularization time was determined to be 24 h.

The optimal decellularization conditions therefore included liquid nitrogen, 0.1% SDS and a decellularization period of 24 h. There were no differences in GAG and collagen content between AF treated with the optimal decellularized method and the native AF group, while the DNA content of the decellularized samples was 85.7% lower than that of the control samples (calculated as [9.1 − 63.55]/63.55).

### Histologic analysis

**Fig. 1:**
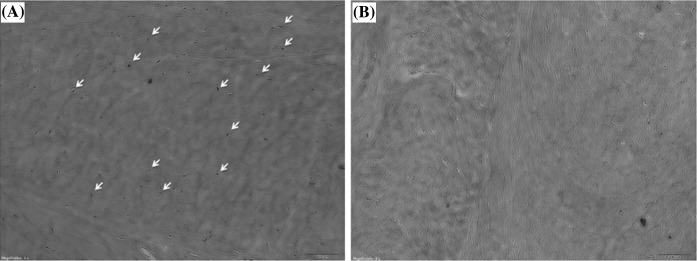
Histology of annulus fibrosus (AF) with H&E stain. Cellular material is **a** clearly embedded in the matrix and **b** rare-to-absent in decellularized AF. Both ×40 magnification

**Fig. 2:**
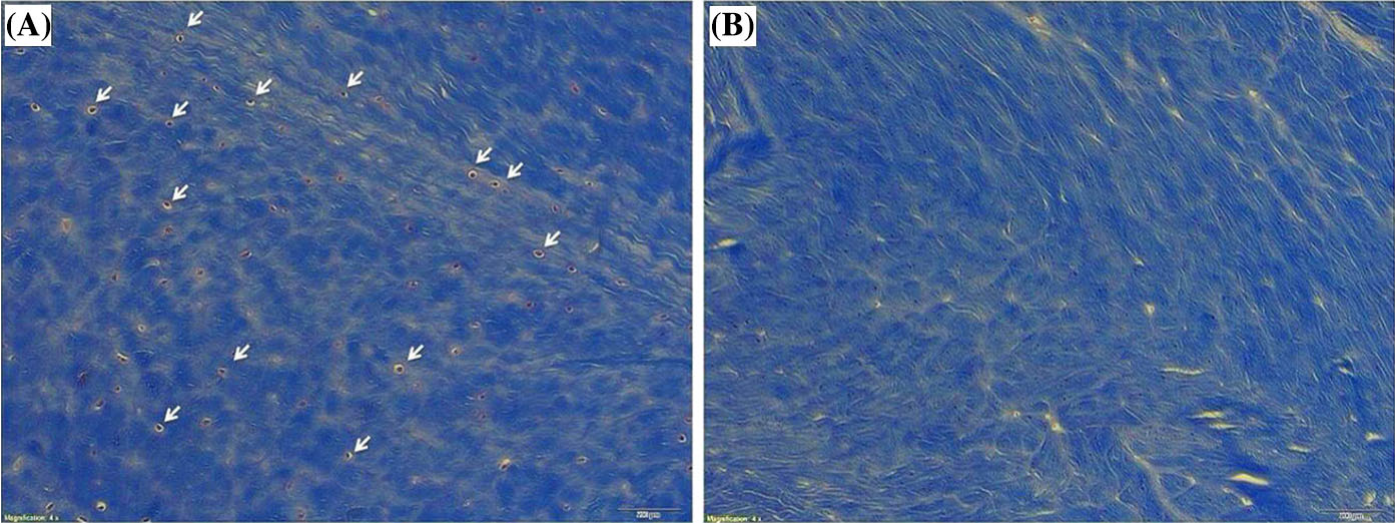
**a** Fresh and **b** decellularized annulus fibrosus stained with Masson’s Trichrome (MT) staining. ×40 magnification

On H&E staining, native AF samples showed many cells to be embedded in the matrix (Fig. 1a). In tissues subject to decellularization under optimal conditions (OC), cellular material was rare on H&E staining (Fig. 1b). On the other hand, MT staining revealed abundant collagen fibrils visible in both native and decellularized AF (OC) without obvious changes in morphology or distribution (Fig. 2). Many cells were seen scattered among collagen fibers in native AF samples (Fig. 2a).

**Fig. 3:**
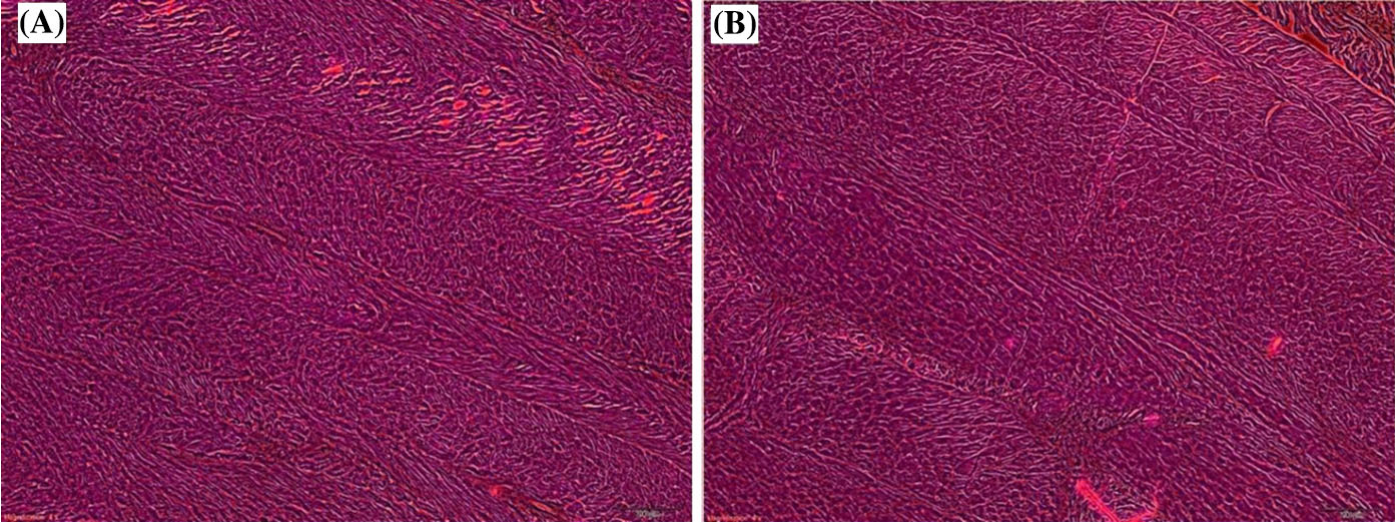
**a** Fresh and **b** decellularized annulus fibrosus stained with Periodic acid-schiff (PAS) staining. ×40 magnification

PAS staining showed that both native AF and decellularized AF (OC) were rich in proteoglycans. Scaffolds prepared by different protocols showed differences in biochemical assays, although histologic assay showed no significant differences, suggesting that the decellularization process did not destroy the microstructure (Fig. 3).

### Scanning electron microscopy (SEM) analysis

**Fig. 4:**
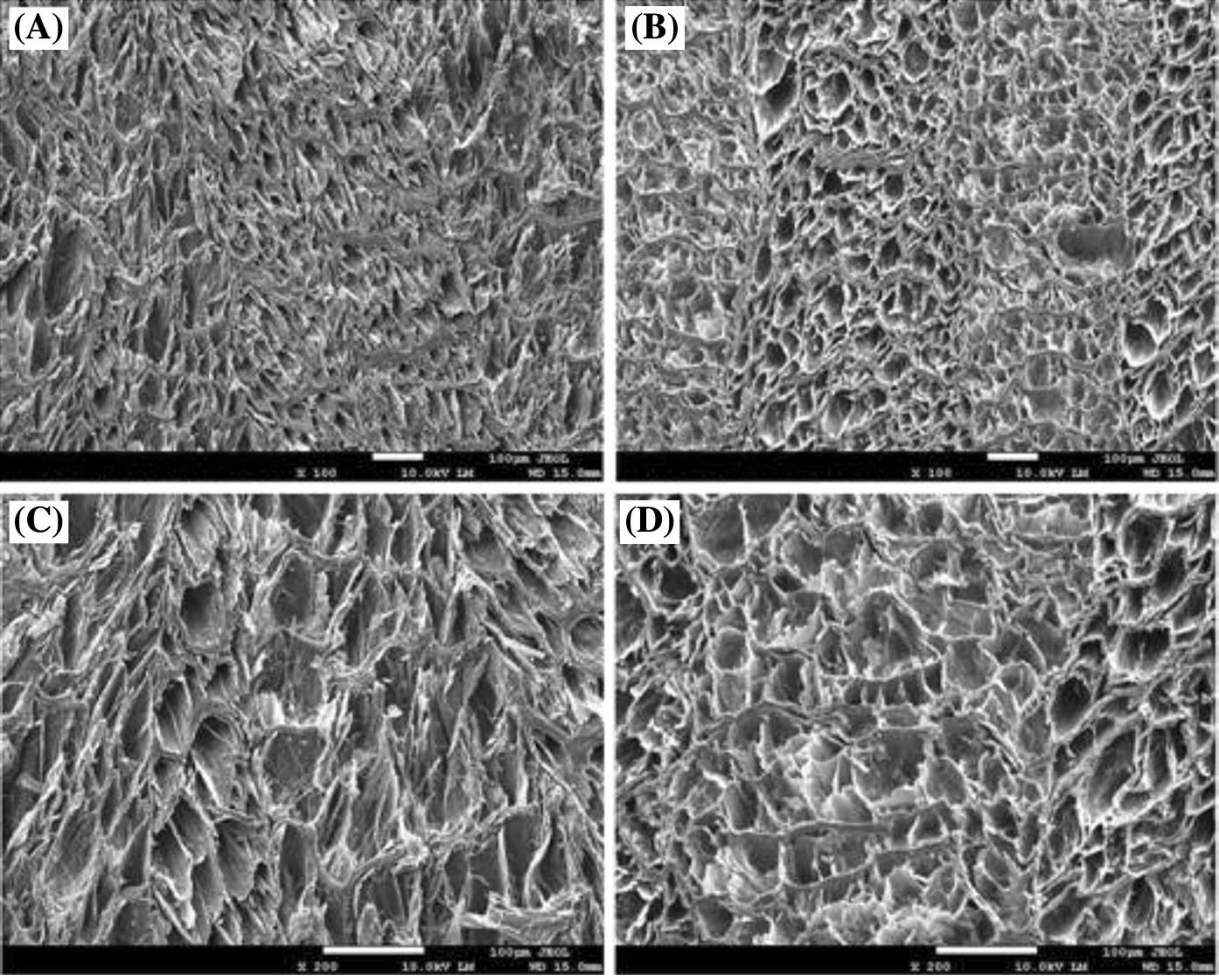
Scanning electron microscopy (SEM) images of annulus fibrosus (AF). **a** Fresh AF has pore-like structures, ×100 magnification. **b** Decellularized AF has confirmed retention of pore-like structures on the AF surface, ×100 magnification. **c** Fresh AF, ×200 magnification. **d** Decellularized AF, ×200 magnification

Scanning electron microscopy (SEM) revealed small pore-like structures on the surface of both native and decellularized AF surfaces (OC), confirming the retention of pore-like structures after decellularization (Fig. 4).

### Biomechanical testing

**Fig. 5:**
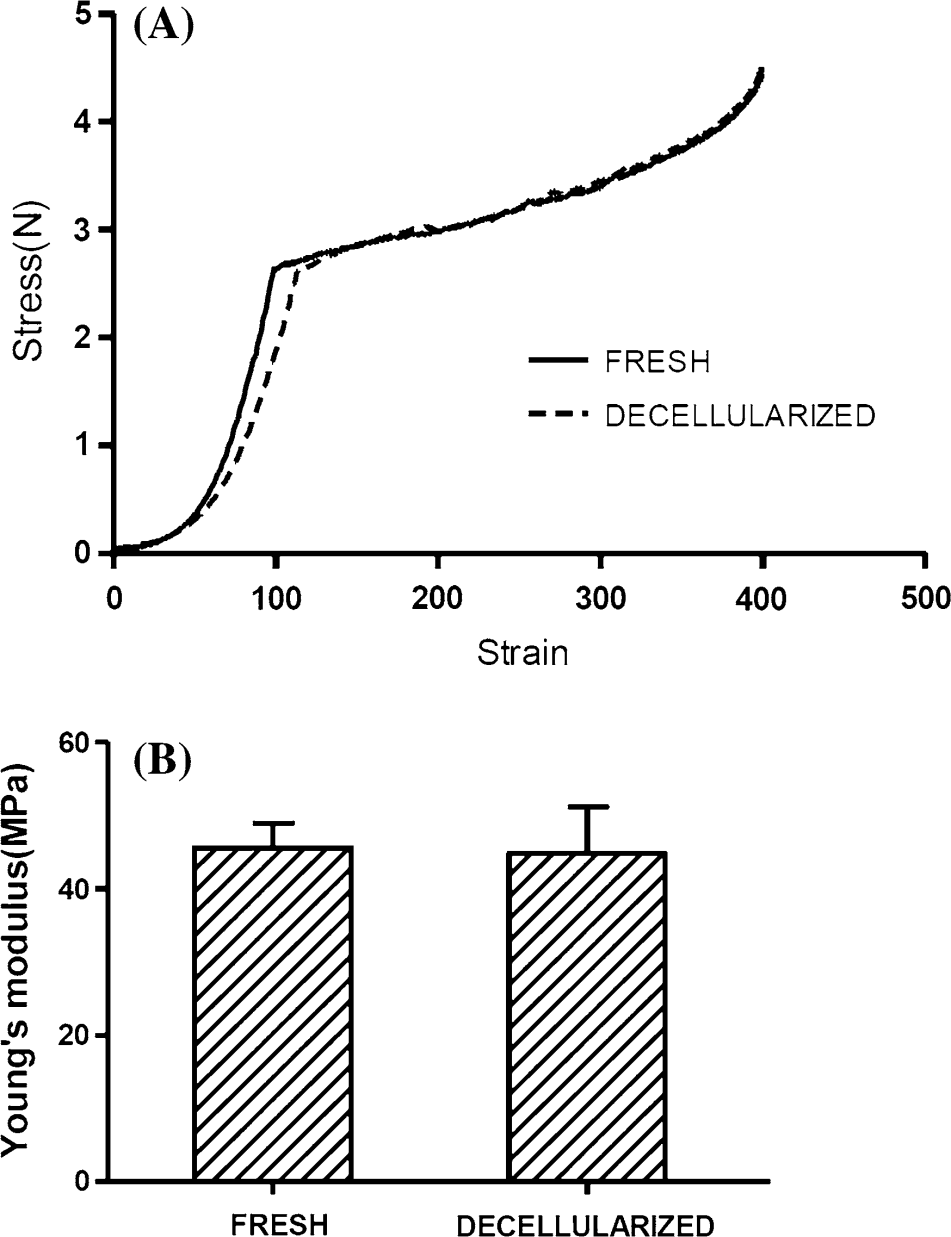
Biomechanical testing of annulus fibrosus. **a** Stress–strain compression curves. **b** Young’s modulus

Using force and deformation results, stress–strain compression curves were constructed and Young’s modulus was obtained (Fig. 5). Compression testing showed no difference between native and decellularized AF scaffolds (*p* > 0.05, Student’s *t* test).

### Cytotoxicity assay

**Fig. 6:**
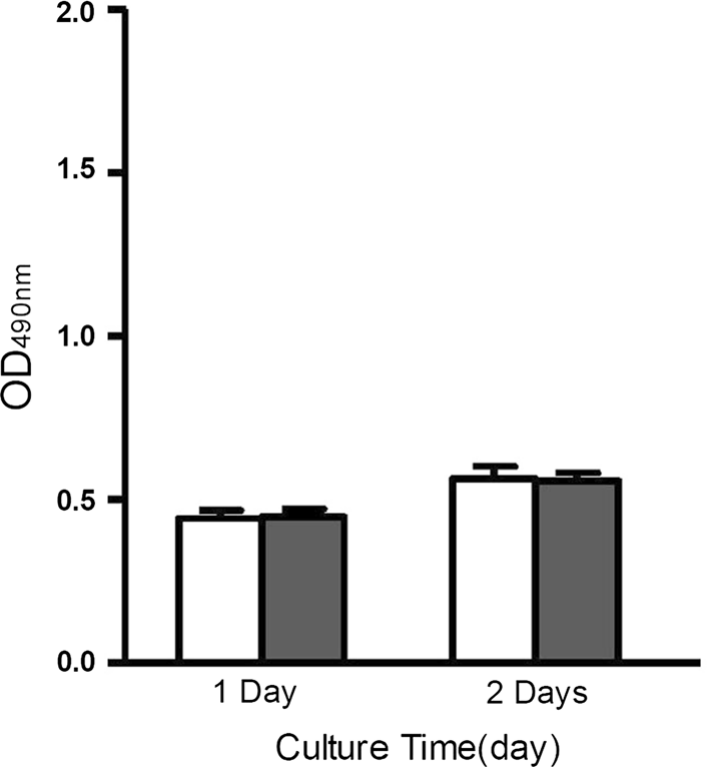
Cytotoxicity studies of the control and decellularized annulus fibrosus (AF). There were no statistically significant differences between decellularized AF and negative control. *Open bar* negative control; *Closed bar* DAF (decellularized AF)

In vitro toxicity studies of control and decellularized AF were conducted using the MTS assay with NIH3T3 cells. At all time points, OD values did not differ between the 2 groups (*p* > 0.05), indicating that the decellularized AF was not cytotoxic (Fig. 6).

### In vivo immuno-compatibility study

**Fig. 7:**
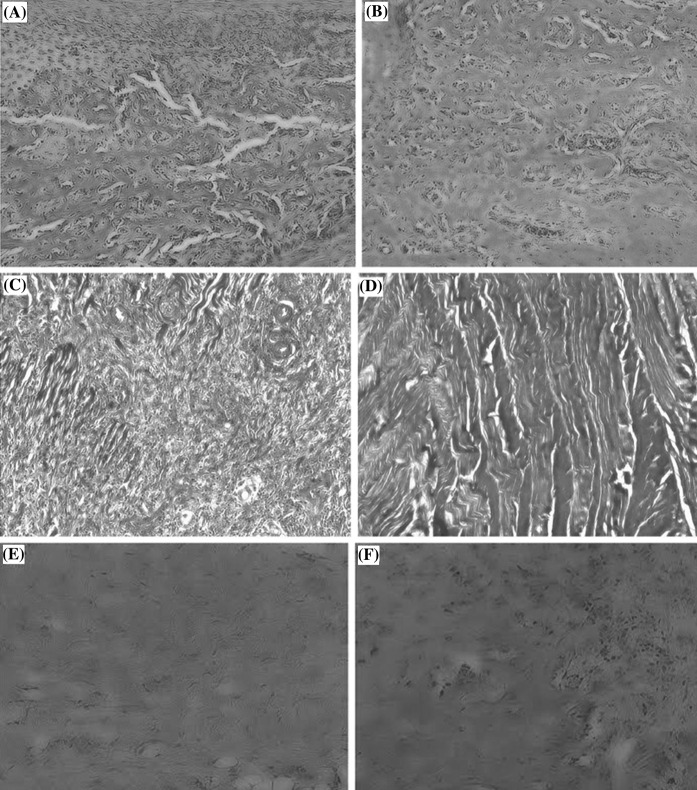
In vivo immuno-compatibility studies at 7 days. **a** Control group with H&E stain, ×40 magnification. **b** Repair group with H&E stain, ×40 magnification. **c** Control group with MT staining, ×40 magnification. **d** Repair group with MT staining, ×40 magnification. **e** Control group with PAS staining, ×40 magnification. **f** Repair group with PAS staining, ×40 magnification

After 7 days of healing, the annulus defect in all samples of both the control and repair groups was covered by scar tissue and populated mostly by mononuclear cells (Fig. 7). MT and PAS staining revealed that the densities of collagen and GAG were higher in the repair group than in controls. There was increased organization of collagen distribution in the repair group compared to the control group.

**Fig. 8:**
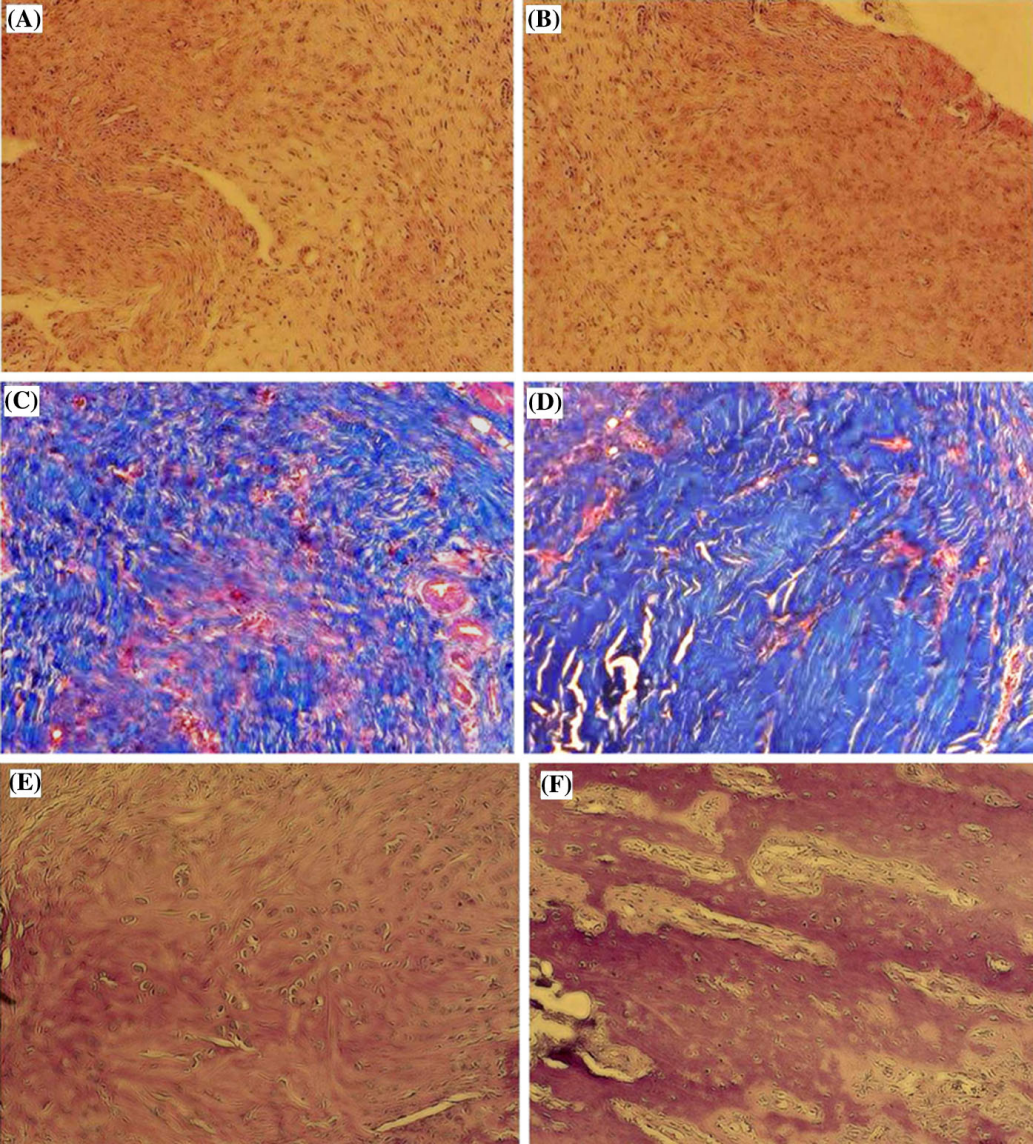
In vivo immuno-compatibility studies at 14 days. **a** Control group with H&E stain, ×40 magnification. **b** Repair group with H&E stain, ×40 magnification. **c** Control group with MT staining, ×40 magnification. **d** Repair group with MT staining, ×40 magnification. **e** Control group with PAS staining, ×40 magnification. **f** Repair group with PAS staining, ×40 magnification

After 14 days of healing, a decrease in mononuclear cells was noted in all samples (Fig. 8). MT and PAS staining revealed that the density of collagen and GAG was still higher in the repair group than in the control group. The density of collagen and GAG was also higher after 14 days of healing than after 7 days. Tissue remodeling was evident at multiple sites, and mononuclear and fibroblast-like cells were found deep within the matrix in both groups.

### Quantification of α-Gal content

**Fig. 9:**
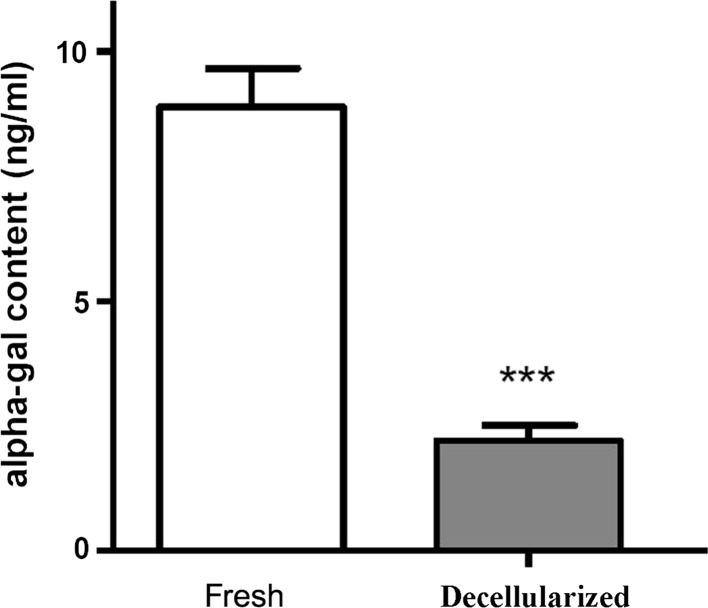
Quantification of α-Gal content. Treatment of annulus fibrosus tissues with the decellularization method resulted in the apparent removal of the α-Gal xeno-antigen (*p* < 0.001)

The α-Gal content was significantly lower in decellularized than in control AF (*p* < 0.001). Treatment of AF tissues under optimal decellularization conditions resulted in apparent removal of the α-Gal xeno-antigen (Fig. 9).

## Discussion

This study investigated optimization of decellularization conditions on AF tissue to identify conditions that maximize retention of ECM properties in the resulting AF scaffold while minimizing toxicity and immunogenic factors such as DNA. Our data suggested that samples decellularized in liquid nitrogen had higher GAG, collagen, and DNA content compared to those decellularized at −80 °C. More GAG and collagen were preserved by decellularization in 0.1% SDS than in Triton X-100, with lower DNA content remaining. A decellularization time of 24 h maximized GAG and collagen preservation while decreasing DNA content relative to that of controls. Results of α-Gal epitope quantitation, in vitro cytotoxicity assays, and an in vivo immuno-compatibility study all showed that decellularization with liquid nitrogen in the presence of 0.1% SDS for 24 h greatly reduced the immunogenicity of porcine AF.

A number of studies have investigated decellularization protocols using different chemical, physical, and enzymatic methods (Dahl et al. 2003; Hongo et al. 2008; Huang et al. 2011; Rieder et al. 2004; Woods and Gratzer 2005). Since any processing steps intended to remove cells could alter the native three-dimensional architecture of the ECM, the goal of most decellularization processes is to minimize disruption, thereby retaining the native mechanical and biological properties of the material. Preliminary studies indicated that decellularization of AF is complex due to its dense collagenous and GAG-rich nature (Gilbert 2012).

Since rapid freezing of a tissue causes formation of intracellular ice crystals, disruption of cell membranes and cell lysis, we compared −80 °C versus liquid nitrogen for physical decellularization. This procedure was followed by chemical treatment to remove cellular material from the tissue. Our data showed that decellularization using liquid nitrogen yielded higher amounts of GAG and collagen compared to freeze–thaw.

Based on their ability to solubilize the cell membrane, ionic detergents such as SDS and non-ionic detergents such as Triton X-100 have been widely studied for tissue decellularization (Crapo et al. 2011; Gilbert et al. 2006). Non-ionic detergents are sometimes more desirable since they have the least impact on protein structure due to their lack of ionic charge, while ionic detergents are considered to be harsher to tissues, with greater disruption of protein structure and loss of matrix components (Gilbert 2012). Early studies reported that SDS was more effective than Triton X-100 for removing nuclei from dense tissues and organs while preserving tissue mechanics (Lumpkins et al. 2008; Nakayama et al. 2010; Pridgen et al. 2011). However, the effectiveness of decellularization using these agents remains unclear due to the variations between different studies in concentrations, temperatures, and times of detergent use. Our present data showed higher retention of collagen and GAG with 0.1% SDS compared to Triton X-100. Decellularization for 72 h in 0.1% SDS effectively removed DNA. However, since there was a higher loss of GAG and collagen compared to the 24 h decellularization protocol, the optimal decellularization time was determined to be 24 h. Our final optimized decellularization method therefore included the use of liquid nitrogen, an ionic detergent (0.1% SDS), and a 24 h incubation period, which yielded the greatest retention of GAG and collagen relative to DNA reduction.

We evaluated the effects of decellularization on the biochemical and biomechanical properties of the resulting scaffold, and whether our decellularization process enabled the retention of collagen and GAG content close to that of the native AF. Our data showed abundant collagen fibrils in both native and decellularized AF, without gross evidence of damage and distribution. There was also no significant difference in collagen content between native and decellularized AF. Our present data showed that 5.6% of GAG was lost during processing, which was an improvement over our previous protocol, which resulted in a 15.9% loss of GAG (Wu et al. 2014).

Tissues were extensively washed in PBS at the end of the protocol since the chemicals and enzymes used for decellularization can be toxic to host cells upon in vivo implantation if they remain within the tissue after treatment. Simple in vitro cytotoxicity tests to examine the biocompatibility of decellularized AF scaffolds indicated that any residual cytotoxic reagents were successfully removed by the washing procedure. In addition, our immune-compatibility data showed that the annulus defects in both control and repair groups in the in vivo study were covered by scar tissue and populated mostly by mononuclear cells on day 7, although the densities of collagen and GAG were higher in the repair group than in controls. Increased organization of collagen distribution in the repair group compared to the control group suggested efficient tissue remodeling in the repair group. Mononuclear cells decreased with time in all samples, while fibroblast-like cell infiltration of the matrix was a key feature of the tissue remodeling observed.

Devising a successful strategy for AF repair requires a clear understanding of the functional biomechanics of the intervertebral disc. Intervertebral discs serve to support large spinal loads involving combinations of tension, torsion, compression, and bending. Therefore, AF repair materials must withstand the high stresses generated by spinal motion (Jin et al. 2013). Biomechanical testing of the decellularized AF demonstrated no significant difference in Young’s modulus, further indicating satisfactory preservation of the biomechanical properties of the native tissue.

Pig-to-human xeno-transplantation offers a potential solution to the shortage of organs for transplantation. However, xeno-transplantation from pigs to humans induces hyper-acute rejection because the α-Gal epitope, expressed in both pigs and humans, is the major antigen inducing complement-mediated cell lysis via human antibodies. Efforts to eliminate the α-Gal epitope from xenografts include (1) the production of alpha1,3-galactosyltransferase knockout pigs (Milland et al. 2005; Puga Yung et al. 2012), (2) treatment of xenogeneic tissue with α-galactosidase (Goncalves et al. 2005; Choi et al. 2012), and (3) introduction of human complement regulatory protein genes into pig cells. Although the cloning of knockout pigs lacking α-Gal epitopes has eliminated the anti-Gal immune barrier to xeno-transplantation, this process is costly. Modification of α-Gal on the cell surface has also been attempted using decellularization. Of the various detergents tested, only SDS is reported to remove these xeno-antigens (Goncalves et al. 2005). Our data suggested that the use of SDS during the decellularization process removed α-Gal xeno-antigens. To the best of our knowledge, our study is the first to report quantification of the α-Gal epitope in decellularized porcine AF tissues.

Residual α-Gal epitopes have been found in commercial bioprostheses of porcine heart valves, SIS-ECM (small intestinal submucosa—extracellular matrix) (Naso et al. 2013; Zheng et al. 2005). Non-α-Gal epitopes can also be immunogenic (Lam et al. 2004; Chen et al. 2005). Previous studies investigating the role of the α-Gal epitope in the host immune response to SIS-ECM reported that the immuno-modulatory effect of the α-Gal epitope did not adversely affect the in vivo remodeling of xenogeneic ECM (Raeder et al. 2002; Daly et al. 2009). Long-term production of anti-non-Gal antibodies against porcine xeno-antigens in human recipients were reported to contribute to a low-level inflammatory process in dense xenografts (Stone et al. 2007).

Most commercially available biologic scaffold materials contain trace amounts of remnant DNA, which is typically present as small fragments and subject to degradation via enzymatic breakdown (Gilbert et al. 2009; Zheng et al. 2005). Although the remaining DNA fragments and α-Gal epitopes are known to stimulate an immune reaction, it is possible that a threshold amount of material is required to adversely affect the remodeling response (Badylak and Gilbert 2008). It is unlikely that any combination of methods will remove 100% of all cell xeno-antigens from a tissue or organ. However, methods that remove most or all of the visible cellular material likely result in biologic scaffold materials that are safe for implantation (Gilbert et al. 2006). Our future work will aim to optimize cell seeding and test the decellularized AF scaffold in a functional large-animal model of disc repair. These studies will determine the in vivo regenerative capacity of porcine AF scaffolds over the long term.

## Conclusions

A porcine decellularization method including freeze–thaw in liquid nitrogen, an ionic detergent (0.1% SDS), and a 24 h incubation period resulted in an AF scaffold which retained the necessary components of the AF matrix, the ultrastructure of the ECM, and the biomechanical properties of the AF. This decellularization protocol also greatly reduced the immunogenicity of porcine AF. The scaffold was therefore biocompatible and is a potential candidate for clinical use.
